# Efficiency of tooth bleaching agent on staining and discoloration characteristics of nicotine stained dental enamel model

**DOI:** 10.1186/s12903-020-01207-2

**Published:** 2020-08-10

**Authors:** Nattha Lertsukprasert, Kitsakorn Locharoenrat

**Affiliations:** Biomedical Physics Research Unit, Department of Physics, Faculty of Science, King Mongkut’s Institute of Technology Ladkrabang, Bangkok, 10520 Thailand

**Keywords:** Absorbance, Enamel, Nicotine, Peroxide, Stain

## Abstract

**Background:**

Surface staining and deeper discoloration characteristics of peroxide-based bleaching agents in the nicotine stain in dental enamel model were evaluated in the present study.

**Methods:**

Nicotine stained dental enamel fragments (*n* = 36) were prepared and were subjected to the bleaching ingredients for a fixed treatment time of 30 min. The bleaching agents were composed of limonene, coconut diethanolamide, and carbamide peroxide served as solvent, nonionic surfactant, and oxidizer, respectively. Optical analysis was carried out considering color stability via colorimeter and UV-Vis spectrometer.

**Results:**

Degrees of color variations were significantly influenced by nicotine content and bleaching ingredient factors. They varied in the range of approximately 3.00 and 5.00 units for all tooth-bleaching agents. The most prominent degrees of color variation elevations were obtained in the tooth bleaching formulae set #2 (1.0% limonene + 20% coconut diethanolamide) in the stained tooth model in comparison to set #1 (0.5% limonene + 10% coconut diethanolamide) and set #3 (1.5% limonene + 30% coconut diethanolamide), partly due to the perceptible color changes. The lowest degree of color variation under a dose limitation was found in the tooth bleaching formulae set #2 + 10% carbamide peroxide formulation. Absorbance spectra were also evaluated after the interaction of bleaching treatment. They confirmed a relationship between nicotine content and discoloration characteristics of the tooth bleaching formulae set #2 + 10% carbamide peroxide.

**Conclusions:**

Carbamide peroxide is considered as generator of free radicals. It converts the color of stains to clear by oxidizing the organic compounds in the stained dental enamel model, achieving whiteness enhancement**.**

## Background

The prolonged color durability of stain molecules including nicotine in e-cigarettes on the dental enamel is considered as one of the important factors included in esthetic requirements [1]. Specific factors such as bleaching time and temperature show the combined and balanced characteristics in capability of a special oral care product to productively clean and clear [2]. Many studies have reported how the treatment time exposed to the bleaching agents tends to increase the stain removal, and how the thermodynamic activity helps the stain bonding to be easily broken apart and residues to be eliminated. Other factors are active component for tooth color stability and base component for tooth cleaning ability. On one hand, the evidence to date suggests that the active component of bleaching agent is composed of hydrogen peroxide of 10–40% for in-office bleaching, or carbamide peroxide of 10–22% (equivalent to 3–7% hydrogen peroxide) for take-home bleaching in order to reduce the stains and change the inherent tooth color. However, effects of current bleaching agents on the dental enamels under the suitable concentrations are still under discussion. De Geus et al. have reported that 10% carbamide peroxide in a gel form gave similar bleaching results in smokers and non-smokers [3]. Oliveira et al. have reported that about 10% carbamide peroxide in a strip form was effectively used for smokers and drinkers [4]. On the other hand, the base agent consist of binding agents (i.e. gum and cellulose derivatives), preventing the split of the contents; humectants (i.e. ethylene glycol and sorbitol), preserving moisture and helping to suspend or dissolve other contents; buffering agents for pH adjustment; and surfactants (i.e. sodium lauryl sulfate) offering high-foaming and surface wetting for debris suspension and isolation. However, none of these studies reported how a combination of limonene and coconut diethanolamide as the base agents and low peroxide concentration is used to reduce the stains and help maintain tooth whiteness.

The theoretical framework for this work is premised on our understanding that the aqueous cleaning technology under the specific bleaching formulation has an impact on the elimination of most enamel stains, with the exception that all orally intact contents needed are all natural products with a small number of chemicals classified by the Food and Drug Administration.

We chose to use of a new bleaching formulation of tooth bleaching agent in a liquid form with carbamide peroxide serving as the main bleaching component, and limonene and coconut diethanolamide serving as the base agents because of two reasons. One is to reduce the chemical commonly usage in the tooth bleaching agent. Two, all ingredients of our base agents are food-grade additives. The hypothesis of this study is that the low foaming and low abrasivity from the base agents are believed to offer true deep stain removal without harming enamel and dentine either with one-off or repeated usage. Specifically, this study aimed to evaluate the physical-chemical characteristics of the carbamide peroxide-based agent together with two natural base agents against color changes in the stained dental enamel model from a long-term usage of e-cigarettes with low-to-high nicotine contents after usage of specific bleaching formulation in a short period as the prolonged outcomes using two techniques- colorimetric and spectroscopic techniques. The colorimeter is used to determine the degree of color changes occurred in each stained tooth/bleaching combinations because the measuring quantitative color alterations with the proper colorimetric techniques have several benefits, such as repeatability, sensitivity, and objectivity [5, 6]. For instance, color values are reported in a CIE L*a*b* system that enabled the interpretation of the degree of color variation (ΔE*) relying on three-color coordinates. L*, a*, and b* indicate the lightness or brightness, greenness (positive) & redness (negative), and yellowness (positive) & blueness (negative), respectively. Numeric information of color grants the exact interpretation of the magnitude of the color difference between objects. The color change implies whether the human eyes observe the differences in overall shades. Therefore, clinical color matching could be estimated according to these values. In addition to the colorimetric technique, staining and discoloration characteristics of tooth bleaching agents are determined using the absorbance spectra acquired via a standard spectroscopic technique, such as UV-Vis spectrometer. After the bleaching treatment, the comparisons color changes of the stained dental enamel model are being carried out in the tooth bleaching formulae set #1 (L0.5 + CD10), set #2 (L1.0 + CD20), set #3 (L1.5 + CD30) with carbamide peroxide of 5–15% and discussed with the literature. L and CD represented limonene and coconut diethanolamide, respectively. The number after the big letter represented the weight per volume (%w/v).

Dental enamel is consisted of hydroxyapatite, which is calcium phosphate of 96% [7]. Calcium phosphate is found to have good mechanical properties in load-bearing bone tissue engineering and its chemical similarity to the mineral in bone. Like dental enamel, calcium phosphate shares the same color, ranging from light-yellow to grayish-white. Even though calcium phosphate is not a perfect match with the dental enamel, the correlations in coatings of calcium phosphate and dental enamel make staining between them comparable. Thus, in this study we use calcium phosphate as the dental enamel model to investigate which nicotine liquids stain dental enamel most.

## Methods

**Fig. 1:**
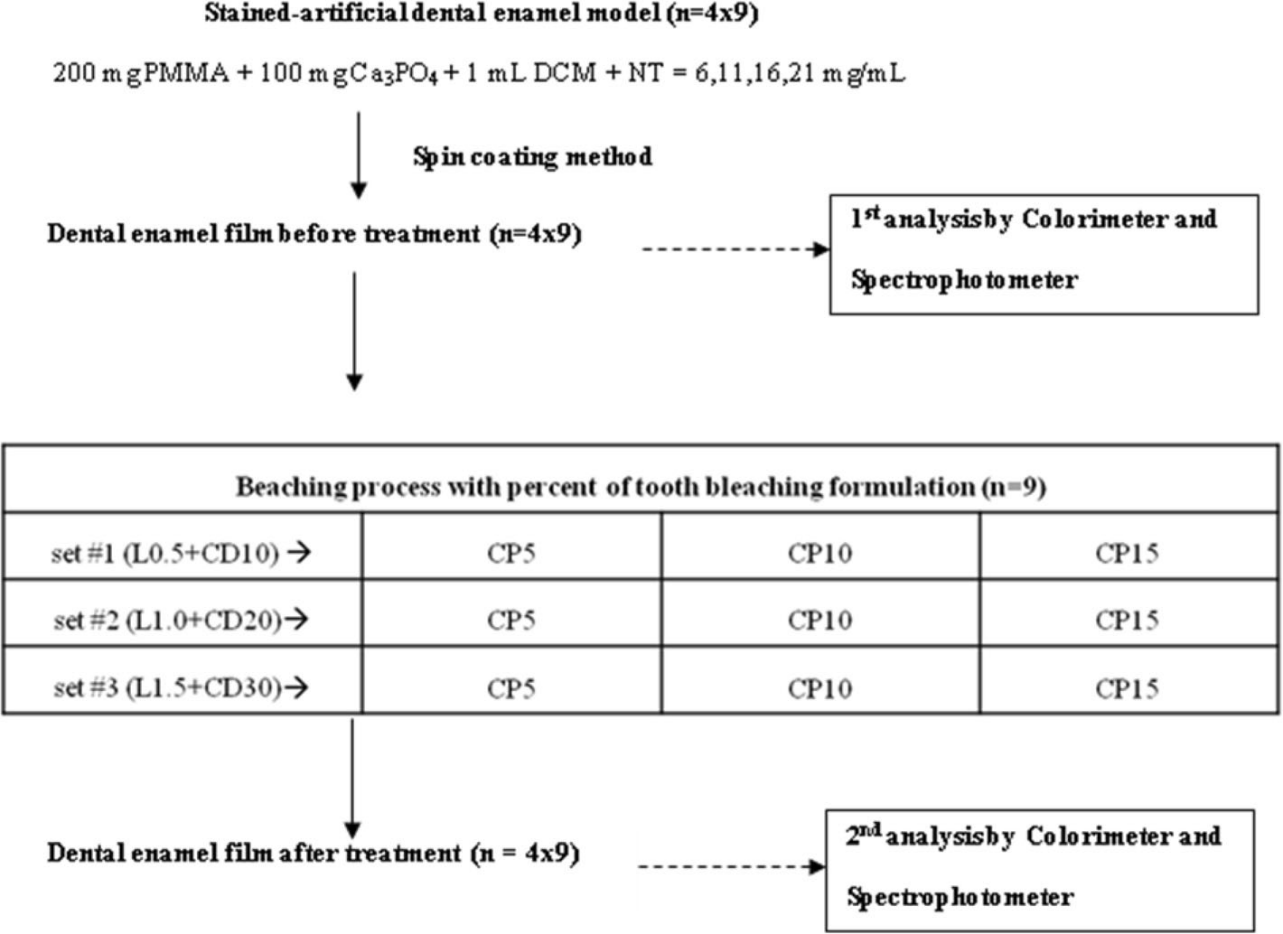
Block diagram of the tooth-bleaching agent in a liquid form for the stained dental enamel model. Percent represents the weight per volume (%w/v) and *n* = 36. The stained enamel specimens of each nicotine dosage were treated with different 1 mL bleaching liquid formulations for a period of 30 min contact duration

Polymethylmethacrylate (PMMA), Ca_3_PO_4_, nicotine, limonene, and carbamide peroxide were purchased from Sigma Aldrich, USA. Coconut diethanolamide was purchased from TSG, Thailand. Dichromethane (DCM) and ethanol were purchased from Sigma Aldrich, USA. In order to achieve the completion of bleaching in as safe a system as possible, slightly lower concentrations of coconut diethanolamide [8], limonene [9], and carbamide peroxide [10] in the liquid tooth bleaching formulae were introduced, as shown in Fig. 1. The ingredient making up each stained-artificial dental enamel model was also represented.

To mimic the smoking action of e-cigarette smoke inside the mouth, calcium phosphate as the dental enamel model was immersed with four different nicotine dosages (NT = 6, 11, 16, 21 mg/mL) for 15 min, resulting in a nicotine level of 6-21 mg/mL comparable to e-cigarettes in the market. This nicotine concentration was also chosen to model the commercially e-cigarettes available in the worldwide market [11]. Then, the dark-colored nicotine solution was mixed with PMMA using DCM as a solvent, and spread on the glass substrate to form a film of stained dental enamel model (thickness = 2.0 μm) via the standard spin coating technique (3000 rpm, 5 s). The first color measurements of each specimen under the dental enamel model with different nicotine contents (6, 11, 16, 21 mg/mL nicotine content) were immediately carried out and recorded with respect to the baseline value (0 mg/mL nicotine content), using a colorimeter (Model: NR200, Hong Kong). The first color measurements were carried out according to the CIELAB system. The color difference ΔE* between two color locations in 3D L*a*b* color area was measured according to the following equation: ΔE* = [(L_a_-L_b_)^2^ + (a_a_-a_b_)^2^ + (b_a_-b_b_)^2^]^1/2^. Seven replicated experiments for each dental enamel model with different nicotine contents were performed so that the mean value was recorded as shown in Tables 1, 2 and 3 (Column #2). We used ANOVA (analysis of variance) for statistical analysis and *p*-value of less than 0.05 implied statistically significant color changes among the tooth bleaching formulae (set #1–3). All the formulae have the same color at the start of the interventions to mimic the mouth. Since our colorimeter does not include a calculation function of the color difference CIEDE2000 (ΔE_00_), ΔE_00_ the up-to-date formula would be compared with the traditional CIELAB (ΔE*). The Excel template code of CIEDE2000 is available in Ref [12].

**Table 1:** Degree of color variation (ΔE* and ΔE_00_) of stained dental enamel model from tooth bleaching formulae set #1 (L0.5 + CD10). L, CD, and CP represented limonene, coconut diethanolamide, and carbamide peroxide, respectively (*p* < 0.05). CP = 10–15%

Nicotine content (mg/mL)	Before treatment		After treatment with tooth bleaching formulae					
	ΔE*	Δ*E*_00_	set #1 + CP5		set #1 + CP10		set #1 + CP15	
			ΔE*	Δ*E*_00_	ΔE*	Δ*E*_00_	ΔE*	Δ*E*_00_
6	4.18	3.78	4.15	3.75	4.03	3.65	3.95	3.59
11	4.43	4.00	4.31	3.88	4.07	3.69	3.90	3.55
16	4.69	4.22	4.51	4.08	4.14	3.75	4.01	3.64
21	4.97	4.56	4.39	3.97	4.17	3.77	3.95	3.61

**Table 2:** Degree of color variation (ΔE* and ΔE_00_) of stained dental enamel model from tooth bleaching formulae set #2 (L1.0 + CD20). L, CD, and CP represented limonene, coconut diethanolamide, and carbamide peroxide, respectively (*p <* 0.05). CP = 10–15%

Nicotine content (mg/mL)	Before treatment		After treatment with tooth bleaching formulae					
	ΔE*	Δ*E*_00_	set #2 + CP5		set #2 + CP10		set #2 + CP15	
			ΔE*	Δ*E*_00_	ΔE*	Δ*E*_00_	ΔE*	Δ*E*_00_
6	4.18	3.78	4.12	3.72	3.87	3.52	3.61	3.32
11	4.43	4.00	4.11	3.71	3.93	3.57	3.62	3.30
16	4.69	4.22	4.19	3.79	4.01	3.63	3.63	3.31
21	4.97	4.56	4.17	3.78	3.99	3.63	3.71	3.39

**Table 3:** Degree of color variation (ΔE* and ΔE_00_) of stained dental enamel model from tooth bleaching formulae set #3 (L1.5 + CD30). L, CD, and CP represented limonene, coconut diethanolamide, and carbamide peroxide, respectively (*p <* 0.05). CP = 10–15%

Nicotine content (mg/mL)	Before treatment		After treatment with tooth bleaching formulae					
	ΔE*	Δ*E*_00_	set #3 + CP5		set #3 + CP10		set #3 + CP15	
			ΔE*	Δ*E*_00_	ΔE*	Δ*E*_00_	ΔE*	Δ*E*_00_
6	4.18	3.78	4.03	3.68	3.92	3.57	3.69	3.36
11	4.43	4.00	4.05	3.69	3.94	3.60	3.74	3.41
16	4.69	4.22	4.11	3.73	3.91	3.57	3.77	3.44
21	4.97	4.56	4.06	3.67	3.90	3.53	3.72	3.40

There was a control group where we used only carbamide peroxide without limonene and coconut diethanolamide. We found that it does not show the real importance of the color change in comparison to the addition of limonene and coconut diethanolamide in a tooth bleaching liquid with carbamide peroxide. A combination of limonene (L), coconut diethanolamide (CD), and carbamide peroxide (CP) in a liquid form was therefore uniformly applied to all stained dental enamel models. That is, the stained enamel specimens of each nicotine dosage were treated with different 1 mL bleaching liquid formulations for a period of 30 min contact duration, which simulated about one bleaching treatment cycle and was used as the time point of color measurement. After the treatment, the second color measurements for each treated specimen were performed. At this point, color evaluations were reported with the same method under the same situations, and in the same manner as earlier explained and reported as the treatment values as shown in Tables 1, 2 and 3 (Column #3–5).

With the same method under the same conditions, staining and discoloration characteristics of tooth bleaching agents for initial color and change in enamel color on the stained dental enamel model were confirmed by absorbance spectra using UV-Vis spectrometer (Model: Avantes-EDU, USA). The bleaching treatments for initial and final color values found for each sample were finally compared in term of the percentage of whiteness with respect to the nicotine content. Also, we used ANOVA (analysis of variance) for statistical analysis and *p*-value of less than 0.05 implied statistically significant color changes among the tooth bleaching formulae (set #1–3). Under seven replicated runs, we used the standard deviation to express how correctly the mean expresses the true nature of the values. Low standard deviation of less than 0.1% indicated that the mean was a fairly reliable expression of the data.

## Results

**Fig. 2:**
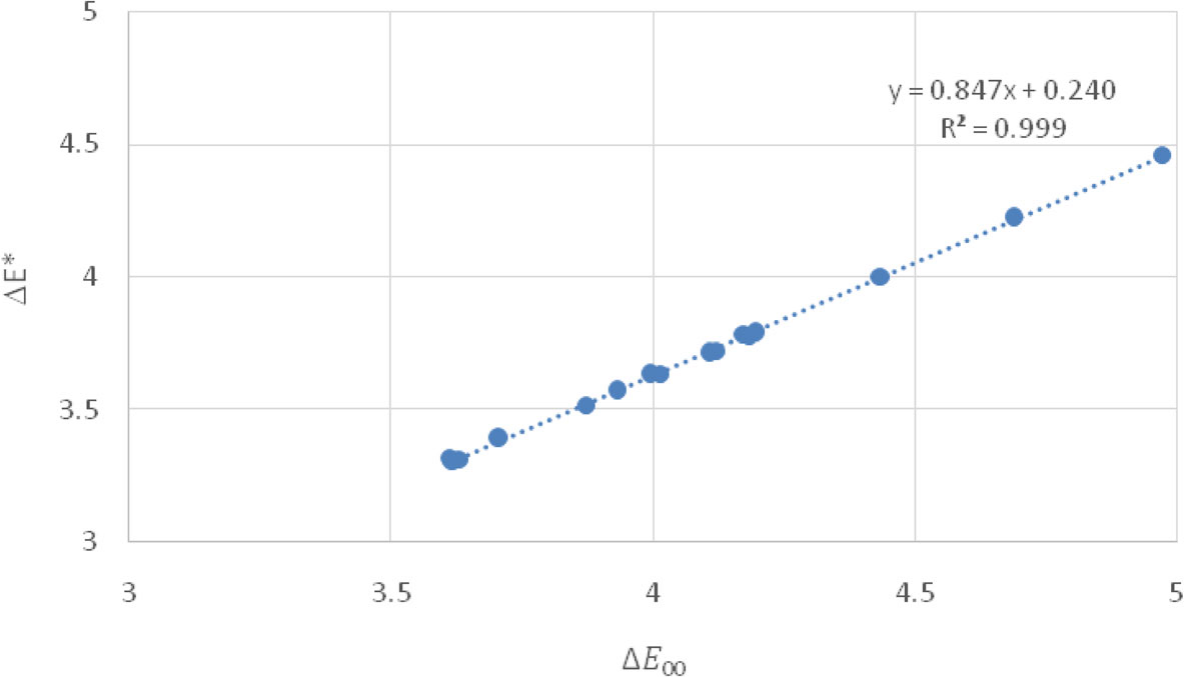
Representative correlation between values of ΔE* and ΔE_00_ of tooth bleaching formulae set #2 (L1.0 + CD20) on the stained dental enamel model

In the present study, the degree of CIELAB color variations varied in a range of 3.61–4.51 units for all bleaching treatments in the stained dental enamel model, as shown in Tables 1, 2 and 3. Note that L, CD, and CP represent limonene, coconut diethanolamide, and carbamide peroxide, respectively. Furthermore, the color difference CIEDE2000 (ΔE_00_) varied in a range of 3.30–4.08 units. A correlation between ΔE* and ΔE_00_ is plotted as a linear fit in Fig. 2. Since CIEDE2000 is an extension of CIELAB with the correction terms of chroma-hue interaction, lightness, chroma, and hue [11], we categorize the tooth bleaching agents into two groups from the CIEDE2000. Any stained tooth/bleaching combinations that resulted in higher than 4.00 units indicated that these bleaching agents could not cause perceptible clinical color changes. However, stained tooth/bleaching combinations that resulted in lower than 4.00 units indicated that these bleaching agents could cause perceptible clinical color changes. Hence, the lowest color change is found in tooth bleaching formulae set #1 (L0.5 + CD10) (ΔE_00_ = 4.08) and the highest is found in tooth bleaching formulae set #2 (L1.0 + CD20) (ΔE_00_ = 3.30) as shown in Tables 1 and 2, respectively.

CP = 10 and 15% for all tooth bleaching formulae exhibits statistically significant discoloration characteristic and shows below 4.00 units. ΔE_00_ values of set #2 (L1.0 + CD20) and set #3 (L1.5 + CD30) are significantly smaller than in set #1 (L0.5 + CD10). Thus, they should show greater color changes. However, set #2 + CP10 applied during one cycle of the treatment could be equally effective and safer for tooth bleaching than to administer treatment session with set #3 + CP15. This less serious color alteration compared to the stained tooth could be related to a usage limitation of limonene and coconut diethanolamide in the bleaching formulae.

**Fig. 3:**
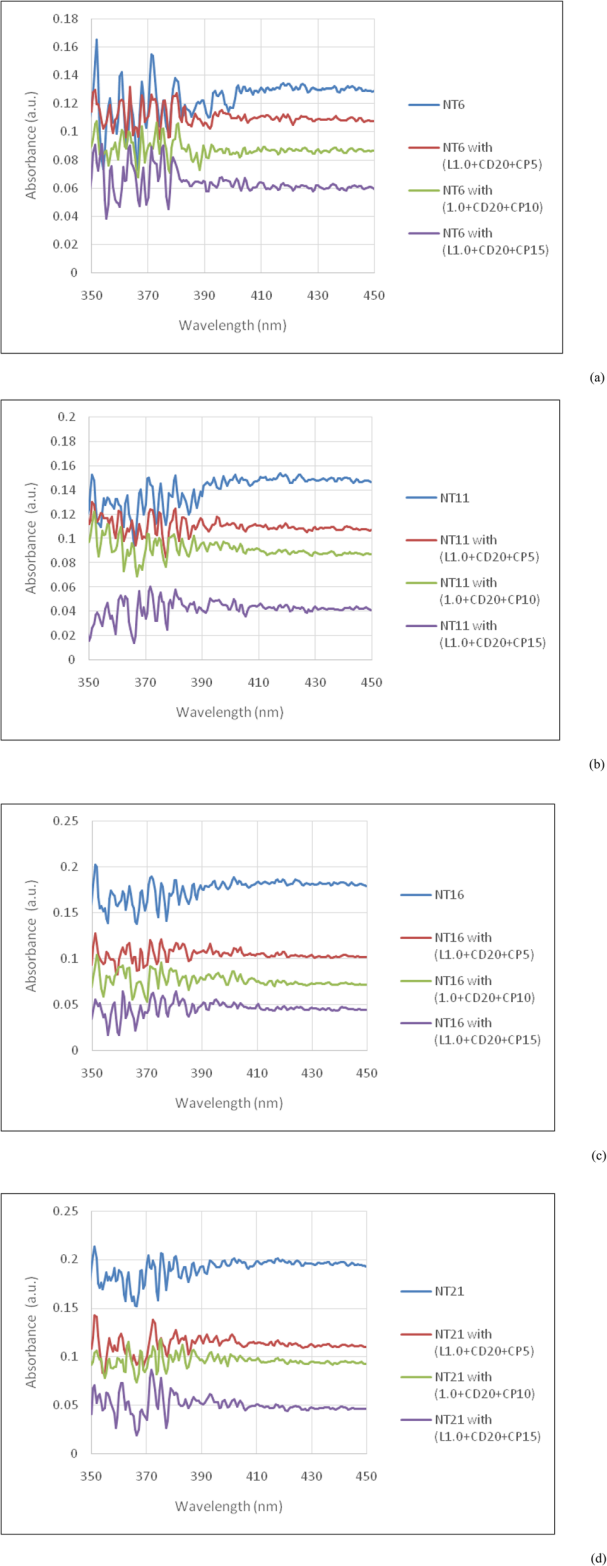
Representative absorbance spectra of tooth bleaching formulae set #2 (L1.0 + CD20) on the stained dental enamel model before and after the tooth bleaching treatment under (**a**) NT6, (**b**) NT11, (**c**) NT16, and (**d**) NT21 representing the nicotine content in 6, 11, 16, and 21 mg/mL, respectively. L, CD, and CP represented limonene, coconut diethanolamide, and carbamide peroxide, respectively

The absorbance spectra of the stained dental enamel model before and after the bleaching treatment are shown in Fig. 3. The calibration curve used to determine the residual nicotine content in the stained dental enamel model after the treatment is Y = 0.004X + 0.102 where Y and X are absorbance peak and nicotine content in mg/mL, respectively. A level of 0 mg/mL of nicotine is used as a reference to compare with the nicotine level of 6-21 mg/mL. It is noted that 0.1 μg /mL of nicotine exposure does not affect the cell viability at 24–48 h exposure; however the cytotoxic effect of nicotine exposure is observed at higher concentrations (10-100 μg/mL at 24 h).

## Discussion

There are a few limitation factors under this study. One, we exclude an influence of the individual salivary on the exact amount of stain removal in stained dental enamel model. Two, rough surface texture between the dental enamel model and restorative materials in dentistry might show a difference in staining susceptibility relating to water absorption rate. Three, this work excludes the pharmacological in vivo studies. Therefore, in vitro model in this study might not mimic the real oral environmental condition. However, the comparative study between in vitro and in vivo will be fully presented in the future work for the possibility of oral care product innovation in the future, as the ethics approval and consent to participate are not applicable under the present study.

As we investigate the color stability between baseline value and 30 min bleaching period from the stained dental enamel model’s layer with a standard spin coating technique, the factors of thickness and surface properties of the stained dental enamel is assumed not to affect the discoloration resistance to the bleaching liquid. Although a testing duration of 30 min is considered as a short period, users are usually recommended to take the bleaching agents, typically daily for at least 1 week depending on the manufacturer’s instructions. However, it should be kept in mind that prolonged durations of taking the bleaching agents would be more problematic regarding the staining efficiencies of nicotine.

**Fig. 4:**
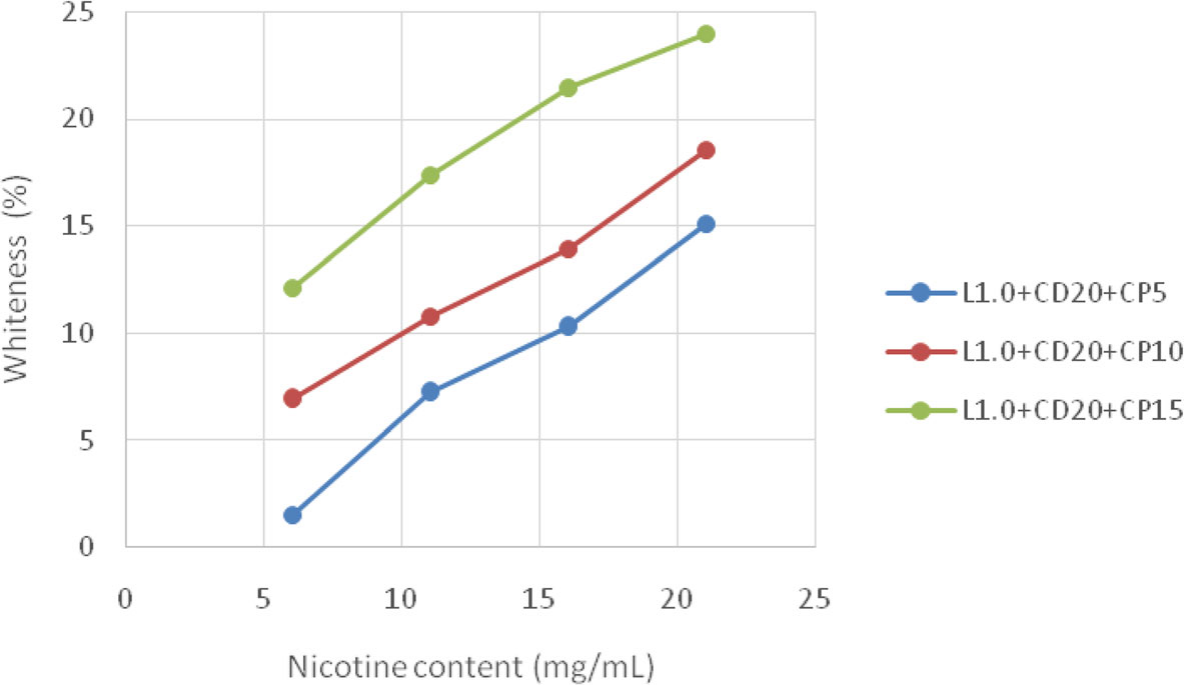
Representative whiteness enhancements from tooth bleaching formulae set #2 (L1.0 + CD20) on the stained dental enamel model. L, CD, and CP represented limonene, coconut diethanolamide, and carbamide peroxide, respectively (*p* < 0.05)

The whiteness of the stained dental enamel model is associated with chemical changes in the materials’ ingredients and water absorption of tooth bleaching agents as shown in Fig. 4. These are related to structural characteristics such as the amount of nicotine in the e-cigarette. The most prominent alteration of color is found in the tooth bleaching formulae set #2 (L1.0 + CD20); conforming to the color change in Table 2, and the lowest is found in the tooth bleaching formulae set #1 (L0.5 + CD10). The tooth bleaching formulae set #3 (L1.5 + CD30) offers the most active discoloration of staining (related to the high content of bleaching agent), but this must be carefully advised because of safety issues with the tooth surfaces [7–9]. Thus, in the present study, tooth bleaching formulae set #2 (L1.0 + CD20) exhibits acceptable color stabilities for all tested bleaching agents. From a calibration curve of nicotine content determination, we verify the remaining nicotine content in the stained dental enamel model from the absorbance spectra in Fig. 3. It is found that about 20% nicotine content remained in the stained dental enamel model treated with set #2 + CP5 from the tooth bleaching formulae set #2 (L1.0 + CD20), whereas the nicotine contents in the stained dental enamel model treated with set #2 + CP10 and set #2 + CP15 are mostly removed. However, set #2 + CP10 is strongly recommended for safety reasons. Furthermore, the content of carbamide peroxide in the present study is in agreement with Ref [3, 4], indicating that our bleaching agent in a solution form is a possible alternative to bleaching agents in gel and strip forms which is both highly safe and systematically effective.

The attractiveness of various dosages of nicotine to the dental enamel model’s surface shows a critical performance in stain removal. Electrostatic and Van der Waals forces act for long periods of the treatment time (30 min), whilst hydrophobic effect, dipole force, and hydrogen bond act for short periods of the treatment time. These actions help the tooth bleaching agents to pre-reach the dental enamel model surface, determining the appearance of the stain removal. The mechanism of action is explained as follows. Carbamide peroxide is moderately stable in anhydrous form and is split into hydrogen peroxide in the aqueous state. For instance, 10% carbamide peroxide is split into 3% hydrogen peroxide and 7% urea. This hydrogen peroxide as an oxidizer produces the superoxide radical as a very reactive oxidizing agent. The free radical then functions by splitting the carbon:carbon double bonds found in the double-bonded organic stains within the dental enamel model’s surface. Splitting these bonds result in the lightening of the molecules until they lose their colors.

Current high-viscosity cleaning and tooth bleaching formulae hold products in intimate contact with the dental enamel model but avoid some degree of penetration of the active ingredients. Hence, we used the appropriate solvent limonene in this study combined with surfactant (coconut diethanolamide), facilitating superior penetration capability and speeding up tooth cleaning and bleaching. The nonabrasive natures of these two substances also remove the possibility of frictional hard or soft tissue damage. In the presence of free radicals, limonene-1-hydroperoxide can form from some unsaturated molecules in limonene. Hydroperoxide is easily decomposed in order to produce free radicals, which accelerate any degradation pathway. Furthermore, a presence of limonene in the tooth bleaching formulae yields an increase in the permeability constant (*K*p) and the flux (*J*). The performance of limonene as a solvent is also inferior to xylene and chloroform [13–15]. Thus, the oxidation rate of limonene accelerates over time. Using limonene in endodontic treatment is becoming well known because of its confirmed biocompatibility, safety, and non-carcinogenic properties.

The most usual surfactant used in bleaching formulae products is the anionic surfactant sodium lauryl sulfate. However, lower-foaming surfactants are efficient and safe to use. Therefore, in the present study, alkanolamide arising from coconut diethanolamide as a nonionic surfactant imparts excellent viscosity, enhancing foam stabilization. It also helps lift stain particles and loosens/suspends the stains from the dental enamel model’s surface via a bubbling action. Its neutralizing nature also avoids rebinding to the substratum, and the bubbling nature assists lifting the stains off the dental enamel model’s surface. Therefore, this substance acts as a good thickening agent and wetting agent. It is also possible that this surfactant is mild on the skin even at high doses and with long-term contact.

## Conclusions

This study attempted to evaluate potential ingredients of tooth bleaching agents on the color stability of nicotine staining in the dental enamel model through colorimetric and spectroscopic techniques. The experimental results have shown that nicotine-stained dental enamel model exhibits significant discoloration values when exposed to the prepared tooth bleaching agents. The limonene, coconut diethanolamide, and carbamide peroxide seemed to be more discoloration active. Considering the bleaching materials, carbamide peroxide = 5% could have less discoloration properties as compared with the composites with different types of tooth bleaching agent. Reduction of nicotine content resulted in increased color stability. All bleaching formulations improved the tooth color, however, carbamide peroxide = 15% produced the most prominent color change as compared with 5 and 10% carbamide peroxide, and whiteness enhancement was higher than the other two. The 1.0% limonene + 20% coconut diethanolamide + 10% carbamide peroxide bleaching formulation had a higher bleaching effect in a similar amount of treatment time and was both highly safe and effective. Upon application of the tooth-bleaching agent, carbamide peroxide breaks down into free radicals. It attaches to the stained portion of the dental enamel model having chromophore bonds. Free radicals tend to break the double chromophore bonds into single bonds. The single bonded C-atom produces the colorless molecules bringing out the natural tooth whiteness. Limonene, the organic soluble element present in the tooth bleaching agents, is able to combine with the peroxide-based bleaching agent, making it insoluble and unable to exert its re-mineralizing property, enhancing the performance of hydrogen peroxide.

## Data Availability

The datasets used and/or analysed during the current study are available from the corresponding author on reasonable request.

## Abbreviations

ANOVA: Analysis of variance
Ca_3_PO_4_: Calcium phosphate
CD: Coconut diethanolamide
CP: Carbamide peroxide
DCM: Dichromethane
Δ*E**: Degree of color variation
h: Hour
*J*: Flux
*K*p: Permeability constant
L: Limonene
min: Minute
mL: Milliliter
mg/mL: Milligram per milliliter
NT: Nicotine
PMMA: Polymethylmethacrylate
rpm: Round per minute
sec: Second
w/v: Weight per volume
μm: Micrometer
